# Inhibitory of active dual cancer targeting 5-Fluorouracil nanoparticles on liver cancer *in vitro* and *in vivo*

**DOI:** 10.3389/fonc.2022.971475

**Published:** 2022-08-05

**Authors:** Mingrong Cheng, Dejian Dai

**Affiliations:** Department of General Surgery, Tianyou Hospital, Tongji University, Shanghai, China

**Keywords:** drug delivery system, liver cancer, nanotechnology, folic acid, chitosan, biotin

## Abstract

The chitosan (CS) material as the skeleton nano-drug delivery system has the advantages of sustained release, biodegradability, and modifiability, and has broad application prospects. In the previous experiments, biotin (Bio) was grafted onto CS to synthesize biotin-modified chitosan (Bio-CS), and it was confirmed that it has liver cancer targeting properties. Single-targeted nanomaterials are susceptible to pathological and physiological factors, resulting in a state of ineffective binding between ligands and receptors, so there is still room for improvement in the targeting of liver cancer. Based on the high expression of folate (FA) receptors on the surface of liver cancers, FA was grafted onto Bio-CS by chemical synthesis to optimize the synthesis of folic acid-modified biotinylated chitosan (FA-CS-Bio), verified by infrared spectroscopy and hydrogen^-1^ nuclear magnetic resonance spectroscopy. The release of FA-CS-Bio/fluorouracil (5-FU) had three obvious stages: fast release stage, steady release stage, and slow release stage, with an obvious sustained release effect. Compared with Bio-CS, FA-CS-Bio could promote the inhibition of the proliferation and migration of liver cancer by 5-FU, and the concentration of 5-FU in hepatoma cells was significantly increased dose-dependently. Laser confocal experiments confirmed that FA-CS-Bio caused a significant increase in the fluorescence intensity in liver cancer cells. In terms of animal experiments, FA-CS-Bio increased the concentration of 5-FU in liver cancer tissue by 1.6 times on the basis of Bio-CS and the number of monophotons in liver cancer tissue by *in vivo* dynamic imaging experiments was significantly stronger than that of Bio-CS, indicating that the targeting ability of FA-CS-Bio was further improved. Compared with Bio-CS, FA-CS-Bio can significantly prolong the survival time of 5-FU in the orthotopic liver cancer transplantation model in mice, and has a relieving effect on liver function damage and bone marrow suppression caused by 5-FU. In conclusion, FA-CS-Bio nanomaterials have been optimized for synthesis. *In vivo* and *in vitro* experiments confirmed that FA-CS-Bio can significantly improve the targeting of liver cancer compared with Bio-CS. FA-CS-Bio/5-FU nanoparticles can improve the targeted inhibition of the proliferation and migration of liver cancer cells, prolong the survival period of tumor-bearing mice, and alleviate the toxic and side effects.

## Introduction

Chitosan (CS) has the advantages of non-toxicity, low immunogenicity, good biocompatibility, and degradability, and has been widely used in the fields of medicine and biomedicine (1). The molecular weight and degree of deacetylation of CS have important effects on its physicochemical and biological properties, and the amino and carboxyl groups on CS also provide the possibility for targeted material modification. Nanocarriers are linked to target targets through ligands to accurately identify and localize tumor cells and tissues (2). The drug carrier targeting function has the following characteristics (3–5): effective for loading anti-tumor drugs; a targeting group that corresponds to the corresponding target cell or tissue; and nanoscale size. As a target cell or tissue, the receptor expressed on it should meet the following two conditions (6): a sufficient amount on the surface of malignant cells and a small amount on the surface of normal cells. So, cell-targeting groups were screened for a variety of tumors and linked to CS nano-drug delivery systems by grafting.

Biotin (Bio), with a molecular weight of 244 Da, is a water-soluble vitamin that binds to its corresponding receptor and exerts corresponding biological functions. Due to the rapidly proliferative nature of malignant tumors, large amounts of vitamins are required during the proliferation process. A large number of biotin receptors are expressed on the surface of tumor cells, so Bio can be used as a tumor targeting ligand to achieve the purpose of targeted drug delivery. The biological functions of Bio in the normal human body mainly include the following aspects (7–9): participate in the metabolism of the three major nutrients; a clear correlation with the metabolism of other vitamins; the metabolism of other nutrients, such as methylation transfer reactions and coenzyme metabolic processes. In biology and medicine, Bio has developed new applications in the past 10 years, such as non-radioactive labeling technology. Bio-nucleic acid probe labeling technology has been developed. There is a trend to replace isotope probes with microspheres, and it has the advantages of economy, safety, reliability, rapidity, and stability; at the same time, it has certain application value in immunoelectron microscopy, radioimmunoassay, and disease monitoring. A large number of Bio receptors are expressed on the surface of malignant tumor cells, and their expression level in liver cancer cells is 39.6 times that in normal liver cells (10), which have a targeted adsorption effect on Bio (11). Our previous experiments found that Bio and CS synthesized biotinylated chitosan (Bio-CS) (12), and found that Bio-CS nanomaterials have strong liver cancer targeting, and its targeting effect is mainly through the nanomaterials. Bio is combined with Bio receptors on liver cancer cells, and the Bio-CS nanoparticles can carry genes *in vivo*, which has an obvious inhibitory effect on the orthotopic liver cancer model, and there are no obvious side effects.

At present, research on drug delivery systems for liver cancer at home and abroad mainly focuses on single-targeted drug delivery systems (13, 14). When a single targeted nanoparticle binds to its corresponding receptor, its binding effect is often affected by a variety of pathological and physiological factors, and sometimes some receptors and ligands fail to bind, resulting in the failure of targeted therapy. For example, in patients with liver disease, the activity and density of the asialoglycoprotein receptor (ASGPR) on hepatocytes is markedly reduced, resulting in a decrease in the number of receptors bound to hepatocytes by approximately 95% (15). Therefore, the active liver targeting effect mediated by a single ASGPR was significantly reduced or even disappeared. In addition, single receptors are not highly expressed in all target cells, resulting in less binding of single-targeted nanoparticles to target cell receptors, resulting in reduced efficacy of targeted therapy (16). There is still room for improvement in the liver cancer targeting of the previous nanomaterial, Bio-CS. The idea of how to reduce the drug concentration in liver tissue and further increase the drug concentration in liver cancer target tissue is an important idea for the transformation of Bio-CS materials. Therefore, our team group proposes to synthesize dual-targeted nanomaterials for liver cancer on the basis of previous experiments, which have shown that they can reduce the drug concentration in liver tissue and increase the drug concentration in liver cancer tissue.

Folic acid (FA) is a small molecule vitamin with no immunogenicity, low cost and easy availability, good stability, and simple and easy chemical bonding with drugs or carriers. Therefore, using FA as a targeting molecule to construct a drug delivery system has become one of the research hotspots in active targeted tumor therapy (17). The FA receptor is a membrane glycoprotein linked to glycosyl-phosphatidylinositol and has a high affinity for FA. As one of the specific receptors that mediate cellular internalization, the FA receptor is capable of uptake of folate into the cytoplasm of eukaryotic cells and is a high-affinity receptor (18). The expression of FA receptor was significantly increased in malignant epithelial tissues and mesothelial tumor cells, and the expression level of FA receptor was not affected by the resistance of tumor cells to chemotherapy (19), based on the difference in FA receptor expression between tumor cells and normal cells, enabling active targeted delivery of FA-drug conjugates (20). After the FA-drug conjugate specifically binds to the FA receptor on the surface of tumor cells, it enters tumor cells through endocytosis. In the weakly acidic environment (pH 5) in the cell, the configuration of the FA receptor changes, the FA-drug conjugate is released, and the receptor can return to the cell membrane surface and then transport other FA molecules or FA-drug conjugates (21, 22). Compared with other macromolecular targeting molecules such as monoclonal antibodies, using FA as a targeting molecule has many unique advantages, such as small relative molecular mass, no immunogenicity, being cheap and easy to obtain, good stability, and compatibility with drug molecules or carriers. The chemical bonding between them is simple and easy, and the targeting application has a wide range.

Therefore, our team group proposed using FA and Bio, dual cancer cell-targeting substances, to synthesize glycosylated chitosan modified by FA (FA-CS-Bio) nanomaterials with CS to solve the defects of single-targeted liver cancer, such as weak targeting and easy targeting failure. In this study, FA-CS-Bio nanomaterials were synthesized with fluorouracil (5-FU) nanoparticles. *In vivo* and *in vitro* liver cancer targeting tests were performed. In terms of animal experiments, the FA-CS-Bio drug-loaded nanoparticles were injected into the tail vein of the mouse orthotopic liver cancer transplantation model, and the 5-FU was transported to the liver through the blood circulation. The Bio and FA on the nanoparticle surface were combined with the corresponding receptors, so that 5-FU can reach the liver cancer tissue smoothly, play the active targeting role of nanoparticle liver cancer, minimize the drug concentration in liver tissue, thereby reducing the toxicity of the drug to normal liver cells, and remove liver cancer in mice, to achieve the purpose of treatment. The results were reported as follows:

## Materials and methods

### Mice and cell lines

The BLAB/C mice, male and female, aged 5-7 weeks and weighing 18–20 g, were raised under a specific pathogen-free humidity and temperature control environment, purchased from the Jiangsu Gempharmatech Co., Ltd. SK-HEP-1, SW480 and normal liver cell line QSG7701 were purchased from the ATCC cell bank in the United States.

### Material synthesis and orthogonal experiment design

Taking the yield of FA-CS-Bio as the investigation index, the factors affecting the synthesis of FA-CS-Bio are the ratio of raw materials, the dosage of catalyst, the reaction time and the temperature during the experiment.


Product yield (PY)(%)=product amount (FA−CS−Bio)/total amount (Bio−CS+FA) 100%

1. Determining the best raw material ratio: Synthesize FA-CS-Bio with FA: Bio-CS in molar ratios of 24:1, 25:1, 26:1, 27:1, and 28:1, and study the effect of different ratios on the production of FA-CS-Bio. The best FA: Bio-CS with the highest PY was selected.
2. Establishment of the optimal catalyst usage ratio: Based on the best FA: Bio-CS, according to FA with 1-Ethyl-3-(3-dimethyllaminopropyl)carbodiimide hydrochloride (EDC.HCl) in the molar ratio of 1:5, 1:6, 1:7, 1:8, and 1:9, FA-CS-Bio was synthesized, and its effect on the PY was observed. The highest best FA: EDC.HCl was selected.
3. Determining the best reaction time: Based on the optimal FA: Bio-CS and FA: EDC.HCl, the reaction times were 6 h, 12 h, 24 h, 48 h, and 72 h, respectively. The best reaction time was selected.
4. Determining the best reaction temperature: The reaction temperatures were room temperature, 46~55°C, 56~65°C, 66~ 75°C and 76~85°C, respectively, based on the optimal FA: Bio-CS, FA: EDC.HCl, and reaction time. The effect of reaction temperature on PY was studied, and the best reaction temperature was selected.
5. The solvent’s influence on PY: Based on the optimal FA: Bio-CS, FA: EDC.HCl, reaction time and temperature, the reaction solvents were divided into dimethylacetamide (DMAc), dimethylformamide (DMF), and dimethyl sulfoxide (DMSO), and the effect of different solvents on the PY was studied.

Taking the PY of FA-CS-Bio as the investigation index, the main factors affecting the synthesis (raw material ratio, catalyst usage, reaction time, and temperature) were analyzed. The orthogonal experiment of L9 (3^4^) was designed according to three different levels, so as to obtain the optimal.

### Fourier transform infrared spectroscopy

The samples of Bio, CS, FA, Bio-CS and FA-CS-Bio were recorded using a Nexus Fourier Transform Infrared Spectrometer (Nicolet™ Natus Medical Incorporated, San Carlos, CA, USA). The infrared spectrometer obtained infrared spectra of samples with five grades. Background readings were taken before each series of measurements. The spectra of the powder samples were scanned at 25°C with 64 scans, the background was removed, and the resolution of the scans was 4 cm^-1^ (23). The spectral range was from 4000 cm^-1^ to 400 cm^-1^.

### Hydrogen^-1^ nuclear magnetic resonance spectroscopy

The Bio, CS, FA, FA-CS, Bio-CS and FA-CS-Bio were tested by hydrogen^-1^ nuclear magnetic resonance spectroscopy (^1^H-NMR, 600 MHz, Agilent Technologies, Santa Clara, CA, USA), and the chemical structures of these final products were observed. The sample was dissolved in a mixed solution of deuterated hydrochloric acid and deuterated water, using tetramethylsilane as the internal standard. The sample was dissolved in a mix of deuterated hydrochloric acid and deuterated water to form a mixed solution. The sample was measured at a frequency of 600 MHz, and the internal standard of the sample was tetramethylsilane (24). And according to the integral area of the characteristic proton peaks of FA, Bio and CS, the degree of substitution (DS) of FA (Bio) was calculated as integrated area FA (or Bio)/integrated area CS×100%.

### Preparation of FA-CS-Bio/5-FU nanoparticles

The FA-CS-Bio/5-FU mass ratio was set to 1:4 (25), and the FA-CS-Bio was placed in a centrifuge tube with an appropriate amount of deionized water and glacial acetic acid (HAC) before being placed in a 50°C water bath. The HAC, deionized water, and 50 mM NaAC were added after heating for more than 10 min to dissolve, and the water bath was placed at 45~50°C for 5~10 min to obtain the FA-CS-Bio solution. The 5-FU was heated to 45~55°C for 5~10 min before being poured into the FA-CS-Bio solution, immediately vortexed (2000 rpm) for 30 s, the sample was lifted for more than 30 min and centrifuged at about 4000 rpm, the residue was washed, dispersed, centrifuged and washed to remove NaOH and 5-FU, etc., and then was redispersed in deionized water, freeze-dried, and saved for later use.

### Particle size analysis and zeta potential determination

The nanoparticle suspension with a 5 mL syringe was sucked and injected into Zeta Potential measurements were taken with a Zetasizer HS3000 (Malvern Instruments, Malvern, UK) in a potential conduit, and particle shapes were determined using a TECNA10 transmission electron microscope (Philips Company, Philips, the Netherlands).

### Preparation of the 5-FU standard curve

The 5-FU standard substance was accurately weighed; the 5-FU concentration with simulated body fluid into a series of standard solutions with concentrations of 0.1, 0.2, 0.5, 1.0, 5.0, 10 and 20 g/mL was prepared, respectively; and 20 mL of the above-mentioned solutions was accurately drawn and injected into a liquid chromatograph (Shimadzu Corp., Kyoto, Japan), and the peak area was measured and recorded according to the chromatographic conditions (26). The regression was performed with the concentration of 5-FU (x) and the corresponding peak area (y).

Conditions used for chromatography: As the stationary phase, an octadecylsilane (C18) column with a particle size of 3.5 µm, an inner diameter of 3.0 mm, and a length of 100 mm was used at 30°C. The mobile phase consisted of acetonitrile, MeOH, and 20 mM ammonium acetate in a volume ratio of 54:36:10 and was used at a flow rate of 1.0 mL/min, with injections limited to 20 L per injection.

### *In vitro* release, encapsulation efficiency, and drug loading assay

20 mg of FA-CS-Bio/5-FU nanoparticles and 5-FU were weighed respectively and put into a dialysis bag. 30 mL of simulated body fluid (SBF) was added to form a suspension (pH 7.4) and shaken at 37°C at a constant temperature. Dynamic dialysis was carried out in the device (frequency of 60 r/min); after 0, 20 min, 40 min, 60 min… (That is, 6 hours), 1 d, 2 d, 3 d… 10 d, the dialysate was taken out, and 30 mL of fresh SBF was added at the same time. The original volume was kept unchanged, and the absorbance value was measured. The amount of 5-FU released by nanoparticles at different times was calculated according to the standard absorption curve (27). The concentration was calculated according to the standard curve equation, and the average value of three experiments was performed. Cumulative drug release (%) = (5-FU released from samples)/(total amount of 5-FU) ×100%

The nanoparticles were destroyed with 1% HCL, SBF was added to the volume, the SBF was used as a blank control, and its absorbance at 254 nm was measured. The encapsulation efficiency and drug-loading capacity of FA-CS-Bio/5-FU nanoparticles were calculated according to the following formula compared to the standard curve of 5-FU in SBF.


Encapsulation efficiency (%)=Drug amount of drug−loaded nanoparticles/Dosage amount×100%

Drug loading(%)=Drug amount of drug−loaded nanoparticles/Total amount of drug−loaded nanoparticles × 100%

### Cell proliferation assay

SW480 and SK-HEP-1 were subcultured in DMEM medium containing 10% fetal bovine serum and dual antibodies (100 U/mL each of penicillin and streptomycin) at 37°C in a 5% carbon dioxide incubator, the tumor cells was collected, centrifuged at 1000 rpm for 5 min, the cell density was adjusted with DMEM medium, and cells in a 96-well plate at 1000 cells/100µL/well was seeded. Each drug (control group, 5-FU, CS/5-FU, Bio-CS/5-FU, FA-CS/5-FU and FA-CS-Bio/5-FU) was fully cultured with DMEM, and formulated into 10, 3, 1, 0.3, 0.1, 0.03, 0.01 µg/mL. Three action time points of 24, 48 and 72 h were selected for each concentration group, and the absorbance was measured at 450 nm with an M5 multifunctional microplate reader (Bio-Rad, Hercules, CA, USA), and each group was detected three times (28). At the same time, the final concentration of 5-FU was 0.3 µg/mL, the cells treated with various treatments, the absorbance was detected 3 times at each time point of 1 d, 2 d, 3 d, 4 d, 5 d, 6 d and 7 d. Tumor inhibition rate = (Control _A450_-Experiment _A450_)/Control _A450_×100%.

### Intracellular drug concentration of hepatocellular carcinoma by FA-CS-Bio

SK-HEP-1 and QSG 7701 cells were cultured. Actively proliferating cells were collected, counted, centrifuged at 1000 rpm for about 5 min, and seeded into culture wells at a concentration of 1000 cells/100 µL. The various drugs (5-FU, CS/5-FU, Bio-CS/5-FU, FA-CS/5-FU, and FA-CS-Bio/5-FU) were prepared in complete medium at 1 µg/mL, 2 µg/mL, 3 µg/mL, 6 µg/mL, and 12 µg/mL incubation time points for each drug concentration. At the same time, each drug group was treated for 1 h, 2 h, 4 h, 6 h, and 8 h with a final concentration of 3 µg/mL of 5-FU. The cells were washed three times, digested with enzymes, centrifuged, the supernatant was removed, 100 µL methanol was added, the cells were freeze-thawed, disrupted, and centrifuged with a high-speed centrifuge, 20 µL of the supernatant was collected, and the intracellular 5-FU concentration was measured by high-performance liquid chromatography according to the pretreatment method, three times in each group. The intracellular drug concentration was calculated from the standard curve. The ratio of the 5-FU concentration in liver cancer cells and hepatocytes reflects the targeting ability of the material to a certain extent. In this study, the ratio of SK-HEP-1/QSG 7701 (S/Q) was used.

### Cell migration assay

50 µL of 5 µg/mL Fibronectin gel (ProSpec-Tany TechnoGene Ltd., Ness-Ziona, Israel) was dropped onto a Transwell chamber (Corning Inc., Corning, NY, USA), and air-dried overnight in a biological safety cabinet. SK-HEP-1 cells were subcultured in DMEM containing 10% FBS at 37°C in a 5% carbon dioxide incubator. SK-HEP-1 cells were collected, counted, centrifuged at 1000 rpm for 5 min, and adjusted the cell density with DMEM; 2000 cells/100 µL/well, seeded cells in the upper chamber, without serum. For the lower chamber, 500 µL of SK-HEP-1 cells containing 10% FBS was collected. In the upper chamber, five drugs (5-FU, CS/5-FU, Bio-CS/5-FU, FA-CS/5-FU and FA-CS-Bio/5-FU) were performed with 0.3 µg/mL of 5-FU. After 48 h of incubation, 50 µL of 0.5% crystal violet solution was added to the upper chamber and photographed. The cells were digested under the chamber with trypsin to make 20 µL of cell suspension. A small amount of the suspension was drawn to fill the counting plate pool, and 5 fields of view under the microscope (the upper, middle, lower, left, and right) were tokenized at 200 magnification and counted through the chamber (28). The number of cells that passed through the membrane was used to evaluate the migration ability, and each group of results was repeated 3 times.

### Animal model establishment

After the mouse model was successfully established by inoculating H22 cells into BLAB/C mice subcutaneously, when the tumor grew to 2~3 cm, the mice were sacrificed, the tumor tissue was dissected out, and the vigorous and fresh tumor tissue was selected to make 6 × 10^7^ cells/mL of tumor cell suspension. After intraperitoneal anesthesia with 20% Ulatan, a median incision was made, and 50 µL of tumor cell suspension was injected into the left hepatic lobe capsule with a 1 mL syringe (29).

### Drug concentration of FA-CS-Bio/5-FU nanoparticles in various tissues

After the orthotopic liver cancer transplantation model was established, they were randomly divided into 5 groups with 3 animal models in each group and were injected into the tail vein with the 5-FU, CS/5-FU, FA-CS/5-FU, Bio-CS/5-FU and FA-CS-Bio/5-FU at doses of 0.5 µg/g, and the mice were sacrificed 24 hours later, and the liver, spleen, kidney, lung, and heart tissues of the animal model were washed with normal saline (blood removed), blotted dry with filter paper, then 0.1 g of tissue was placed in a centrifuge tube, and 1 mL of 50% methanol was added. The cells were crushed into a homogenate with an ultrasonic cell crusher and centrifuged at 8000 r/min for 10 min (30). 20 µL of the supernatant was aspirated, and the concentration of 5-FU in each tissue was measured by high performance liquid chromatography.

### Laser confocal detection

SK-HEP-1 cells and QSG 7701 cells were seeded in 6-well plates, incubated for 24 h, and then fresh medium containing different FITC-labeled CS, FA-CS, Bio-CS, and FA-CS-Bio nanoparticles was incubated for 4 h. Thereafter, after fixing cells with 4% paraformaldehyde for 20 min at room temperature, the nuclei were stained with the fluorescent dye Hoechst 33258 (Biyuntian Biotechnology Co., Ltd., Shanghai, China), and washed three times with 0.01 M PBS. The coverslip was taken out with small tweezers and placed on a glass slide. After mounting with glycerol buffer solution, the fluorescence images of the samples were observed by a laser co-polymerization microscope (Olympus FV-1000, Tokyo, Japan) (31). Laser excitation wavelength during scanning: FITC was 488 nm, Hoechst 33258 was 405 nm, Alexa was 633 nm, and the captured images were superimposed with NIS element imaging software.

### Dynamic imaging of FA-CS-Bio nanoparticles *in vivo*

In order to ensure that the fluorescence signal intensity of different modified nanoparticles was consistent, some amino groups on the nano-framework CS with Rhodamine B Isothiocyanate (RBITC) were reacted to obtain the same amount of isothiocyanate. Rhodamine B-labeled nanomaterials RBITC-CS, and then synthesized RBITC-Bio-CS, RBITC-FA-CS, and RBITC-FA-CS-Bio. On the fifth day after the orthotopic liver cancer transplantation model in mice, RBITC-CS, RBITC-Bio-CS, RBITC-FA-CS, and RBITC-FA-CS-Bio nanoparticles were injected from the tail vein at a dose of 0.4 mg/20 g. In mice, the dynamic distribution of CS, Bio-CS, FA-CS, and FA-CS-Bio in mice was dynamically observed by a small animal *in vivo* imaging system (CRi Maestro™, USA). The observation time points were 2 h and 4 h, 8 h, 12 h, and 24 h, respectively. Three mice in each group were sacrificed at each time point, and the fluorescence intensities in liver, kidney, spleen, and brain tissues were detected, representing the amount of nanoparticles entering the site. After the mice were sacrificed at the time point of 24 h, the number of fluorescent photons in the liver and liver cancer areas was detected by the CRi Maestro™ imaging system (32), and the fluorescence ratio of liver cancer (C)/liver (L) was calculated.

### Survival analysis of FA-CS-Bio/5-FU nanoparticles in the mouse orthotopic liver cancer transplantation model

On the 5th day after the mouse orthotopic liver cancer transplantation model was established, the liver cancer tissue was about 4~6 mm, and the experimental model was divided into 6 groups: control, FA-CS-Bio, 5-FU, CS/5-FU, FA-CS/5-FU, Bio-CS/5-FU and FA-CS-Bio/5-FU groups. The control group received an intravenous injection of normal saline. The dose was 200µL. FA-CS-Bio group: 200 µL was intravenously injected with FA-CS-Bio. 5-FU, CS/5-FU, FA-CS/5-FU, Bio-CS/5-FU, and FA-CS-Bio/5-FU groups were respectively given corresponding drugs in 200 µL (containing 0.371 mg of 5-FU). Beginning on the 6th day after molding, administration through the tail vein continued for 5 days. Nine experimental model mice in each group were used for survival analysis.

### Blood biochemical analysis

After 10 days of treatment, the serum samples of the mice in each group were taken, and the blood biochemical indexes alanine aminotransferase (ALT) and aspartate aminotransferase were detected by the DA 3500 Discrete Analyzer automatic chemical analyzer (Fuji Medical System Co., Ltd., Tokyo). The white blood cells (WBC), red blood cells (RBC), hemoglobin (Hgb), and platelets (PLT) were detected by the Sysmex XS-800i automatic blood cell analyzer (Sysmex Shanghai Ltd, Shanghai, China).

### Statistical analysis

Normally distributed data are expressed as mean ± standard deviation, A *t*-test was used to compare the two groups; an one-way analysis of variance (ANOVA) was used to compare multiple groups; and the least significant difference method (LSD-*t*) was used for pairwise comparison between groups. Survival time was analyzed by the Kaplan-Meier method, and *p<*0.05 was regarded as statistically significant.

## Results

### Optimized synthesis of FA-CS-Bio materials

It can be seen from **Table 1** that when the molar ratio of FA: Bio-CS was from 24:1 to 26:1, the PY of synthesizing FA-CS-Bio gradually increased, while its PY gradually decreased from 26:1 to 28:1, and the optimal FA: Bio-CS molar ratio was 26:1. On the basis of the synthesis molar ratio of FA: Bio-CS of 26:1, the PY of FA-CS-Bio synthesized from the molar ratio of FA: EDC.HCl gradually increased from 1:5~1:8, while its PY appeared to have gradually decreased from 1:8~1:9, and its optimal FA: EDC.HCl molar ratio was 1:8; based on the synthesis molar ratios of FA: Bio-CS and FA: EDC.HCl were 26:1 and 1:8, respectively. FA-CS-Bio’s PY increased significantly when the reaction time was from 6 h to 24 h, and its PY decreased significantly when the reaction time was from 24 h to 72 h, indicating that the optimal reaction time was 24 h; the molar ratios of FA: Bio-CS and FA: EDC.HCl were 26:1 and 1:8, respectively, and the reaction time was 24 h to synthesize FA-CS-Bio. The PY was the best at room temperature, and the PY decreased significantly with the increase in temperature; there was no significant difference in the PY of FA-CS-Bio synthesized in DMAc, DMF, and DMSO solvents.

**Table 1:** Analysis of influencing factors on FA-CS-Bio material synthesis.

FA : Bio-CS (mol:mol)	PY (%)	FA: EDC.HCl (mol:mol)	PY (%)	Reaction time (h)	PY (%)	Temperature (°C)	PY (%)	Solvent	PY (%)
24:1	11.43	1:5	19.83	6	8.42	Room temperature	24.42	DMAc	24.75
25:1	19.92	1:6	20.42	12	21.82	45~55	14.25	DMF	24.42
26:1	23.75	1:7	23.53	24	24.10	55~65	17.32	DMSO	24.93
27:1	23.08	1:8	24.18	48	23.54	65~75	16.82	–	
28:1	21.75	1:9	23.31	72	23.07	75~85	14.52	–	

Taking the synthetic yield of FA-CS-Bio as the investigation index, the ratio of synthetic raw materials, catalyst usage ratio, reaction time, and temperature were studied. Each factor was designed according to three levels, and the L9 (23) experimental scheme was designed, and the following process was formulated (**Table 2**).

**Table 2:** Synthetic Bio-GC orthogonal experiment design.

Levels	FA : Bio-CS (Mol : Mol)	FA: EDC.HCl (Mol : Mol)	Reaction time (h)	Temperature (°C)
1	25:1	1:7	12	Room temperature
2	26:1	1:8	24	45~55
3	27:1	1:9	48	55~65

1 g of FA-CS-Bio was accurately weighed and dissolved in DMSO solvent. An orthogonal test was performed according to **Table 3**. After synthesizing FA-CS-Bio, the yield was calculated and optimized according to the yield. It is now known that R represents the range analysis value of three levels, and the larger the value, the greater the influence of this factor on the yield of synthetic Bio-GC. It could be seen from **Table 2**.9 that the range analysis shown that the influence factors on the PY of FA-CS-Bio was B > D > A > C, that was, the influence factors on the yield of FA-CS-Bio were FA: EDC. HCl, temperature, FA : Bio-CS and reaction time in descending order. From the analysis of the average yield in different levels, it could be seen that the optimal conditions for the synthesis of Bio-GC were A_2_B_2_C_2_D_1_ as the best yield, that was, when FA : Bio-CS was 26:1, FA : EDC.HCl was 1:8, and the reaction time was 24 h, the PY of FA-CS-Bio was the highest when the reaction was carried out at room temperature.

**Table 3:** The experimental results of orthogonal test arrangement.

NO.	FA : Bio-CS(A)	FA: EDC.HCl(B)	Reaction time(C)	Temperature(D)	YP(%)
1	1	1	1	1	20.83
2	1	2	2	2	19.42
3	1	3	3	3	12.54
4	2	1	2	3	21.62
5	2	2	3	1	24.42
6	2	3	1	2	16.02
7	3	1	3	2	19.72
8	3	2	1	3	21.42
9	3	3	2	1	20.31
K1	17.60	20.72	19.42	21.85	
K2	20.69	21.75	20.45	18.38	
K3	20.48	16.29	18.89	18.53	
R	3.09	5.46	1.56	3.47	

The average yield of FA-CS-Bio synthesis at different levels for each factor of K1, K2 and K3; R is the difference between the maximum average yield and the minimum yield of each factor at the three levels.

Verification of the optimized synthesis conditions by Bio-GC: FA and EDC.HCl was dissolved in DMSO at a molar ratio of 1:8 in the dark at 25°C, and mixed with magnetic stirring for 3 h to obtain an FA solution. Then continue to add Bio-CS with a molar ratio of FA : Bio-CS of 26:1 to the above solution slowly under magnetic stirring, and reacted at room temperature for 24 h. After the reaction, the reaction solution was moved to a dialysis bag and dialyzed with distilled water for 3 d. The water was changed every 6 h, and FA-CS-Bio was obtained by freeze-drying, and its PY was 23.76%.

### Infrared spectroscopy and ^1^H NMR spectroscopy of FA-CS-Bio materials

**Figure 1:**
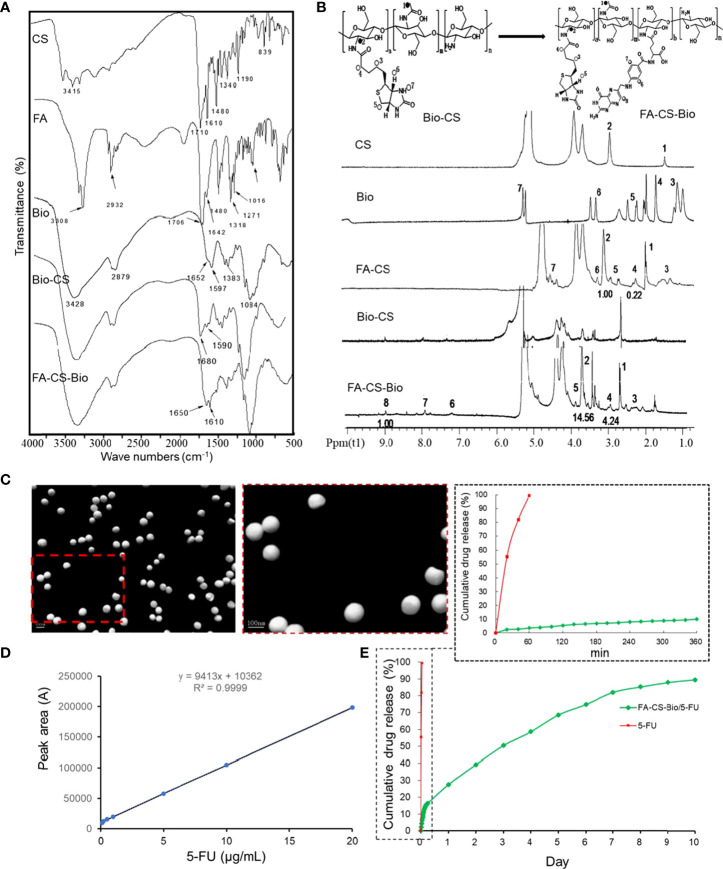
Synthesis and sustained release of FA-CS-Bio nanomaterials **(A)** Fourier transform infrared spectra of different nanomaterials; **(B)** ^1^H NMR of different nanomaterials; **(C)** Transmission electron microscope image of FA-CS-Bio/5-FU nanoparticles; **(D)** Preparation of 5-FU standard curve; **(E)** *In vitro* release of FA-CS-Bio/5-FU nanoparticles.

It can be seen from **Figure 1A** that the FA-CS-Bio material was mainly obtained by grafting FA and Bio on its main chain by amidation of CS. It can be seen from the infrared spectrum that there were two characteristic absorption peaks of the amide bond of CS at 1597 cm^-1^ and 1652 cm^-1^; the infrared spectrum of Bio-CS shew that 1597 cm^-1^ of chitosan corresponded to the amino group (-NH2) in the bending vibration of the N-H bond, on the Bio-CS spectrum, the peak at 1590 cm^-1^ was greatly weakened, while the 1680 cm^-1^ corresponded to the stretching vibration of the carbon-oxygen double bond (C=O) in the amide bond. The absorption peak was greatly enhanced, so it can be proved that Bio was successfully coupled to CS through the amide bond. The absorption peaks of the amide I band and the amide II band corresponding to Bio were shifted and the peak shapes were changed, which fully indicated that amidation reaction occurred on the amino group of the chitosan molecular chain, and both Bio and FA were coupled connect.

It can be seen from **Figure 1B** that the vibration peaks at 1 and 2 on the CS nuclear magnetic spectrum correspond to the protons in its molecular structure diagram; for Bio, 3 (4.99 ppm), 4 (5.69 ppm), 5 (6.60 ppm), 6 (7.63 ppm), and 7 (9.98 ppm) were the vibrational peaks of protons in the molecular structure, respectively, and the vibrational peak at 8 (15.63 ppm) was attributed to the carboxyl proton hydrogen at the end of the five-carbon chain. Bio was activated by NHS and DCC, and participated in after the amidation reaction; the carboxyl hydrogen no longer existed, so there was no proton vibration peak associated with it at the corresponding chemical shift in the Bio-CS hydrogen spectrum of the product. There were characteristic peaks corresponding to protons in Bio molecular structures such as 3, 4, 5, 6, and 7 at 3.22, 4.47 ppm, etc., respectively. As for the peaks corresponding to protons at 1 and 2 of the CS spectrum, they appeared in chemical shift at 1.94 and 3.04 ppm. The NMR peaks of the synthesized material Bio-CS indicated that Bio was successfully coupled to CS to obtain Bio-CS. It can be seen from **Figure 1B** that the characteristic peaks 1 and 2 on the FA-CS-Bio hydrogen spectrum were assigned to the corresponding protons in CS, of which the integral area of ​​1 was 14.56, and the characteristic peaks 3, 4, and 5 were assigned to Bio molecules, of which the integral area of ​​4 was 4.24. The NMR peaks of the synthesized material Bio-CS indicated that Bio was successfully coupled to CS to obtain Bio-CS with a DS of 29.12% (4.24/14.56). The proton vibration peaked in the structure, the characteristic peaked at 6 (7.2 ppm), 7 (7.9 ppm), and 8 (8.9 ppm) correspond to protons in three different chemical environments in the FA molecule, of which the integral area of ​​8 was 1. The NMR peaks of the synthesized material FA-CS-Bio indicated that FA was successfully coupled to Bio-CS to obtain FA-CS-Bio with a DS of 6.87% (1/14.56). From the above-mentioned infrared spectroscopy and nuclear magnetic determination of the material, combined with the spectral analysis, it was fully shown that the FA-CS-Bio material was successfully synthesized.

### Synthesis and characterization of FA-CS-Bio/5-FU nanoparticles

It can be seen from **Figure 1C** that FA-CS-Bio/5-FU nanoparticles were shown by the electron microscope: FA-CS-Bio/5-FU nanoparticles were spherical, with smooth surface, uniform size, good dispersion, and no agglomeration; the particle size was 80.7 nm, the Zeta potential was 16.4 mV, the encapsulation efficiency was 81.5%, and the drug loading was 14.68%.

It can be seen from **Figure 1D** that the 5-FU concentration had a good linear relationship with the peak area between 0.1 and 20 mg/L, and the regression equation was y=9413x+10362 (r=0.9999). It can be seen from **Figure 1E** that the cumulative release rate of 5-FU in the simulated body fluid reached 96.5% within 1 h, showing a near-linear rapid release, while the release of FA-CS-Bio/5-FU nanoparticles had three distinct stages: Rapid release stage: the time was 0~6 h, the cumulative release rate was 16.41% due to the rapid diffusion of the drug on the surface of the nanoparticle or the superficial layer of the nanoparticle into the simulated body fluid; steady release stage: the time was 7 h~7 d, Its release was steady, the cumulative release rate was 82.11%, and the cumulative release rate increased by 65.7% during this period; slow release stage: the time was 8 d~10 d, this stage may be related to the complete degradation of nanomaterials, the cumulative release rate was 89.56%, and the cumulative release rate increased by 7.45% in this stage.

### FA-CS-Bio/5-FU nanoparticles inhibit the proliferation of hepatocellular carcinoma cells

**Figure 2:**
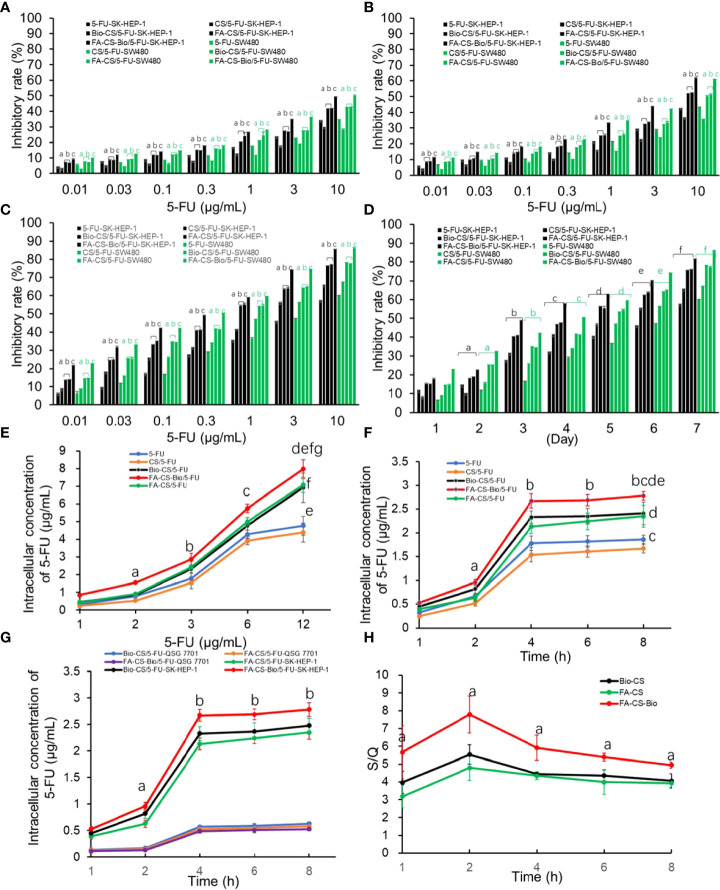
Effects of different 5-FU nanoformulations on the proliferation and intracellular 5-FU concentration of liver cancer cells **(A)** After 24 hours of culture, the effects of various 5-FU nanoformulations on tumor cell proliferation Note: compared with 5-FU, ^a^ *p* ^<^0.01; compared with CS/5-FU, ^b^ *p* ^<^0.01; compared with FA-CS/5-FU or Bio-CS/5-FU, c*p*<0.01. **(B)** The effects of various 5-FU nanoformulations on tumor cell proliferation after 48 hours of culture. Note: compared with 5-FU, a*p*<0.01; compared with CS/5-FU, b*p*<0.01; compared with FA-CS/5-FU or Bio-CS/5-FU, c*p*<0.01. **(C)** The effect of different 5-FU nano-formulations on tumor cell proliferation after 72 hours of culture. Note: compared with 5-FU, a*p*<0.01; compared with CS/5-FU, b*p*<0.01; compared with FA-CS/5-FU or Bio-CS/5-FU dosage form, c*p*<0.01. **(D)** The effect of different 5-FU nano-formulations on the proliferation of tumor cells cultured for 1–7 days at a 5-FU concentration of 0.3 g/mL. Note: compared with cultured 1 d, a*p*<0.01; compared with cultured 2 d, b*p*<0.01; compared with cultured 3 d, ^c^ *p* ^<^0.01; compared with cultured 4 d, ^d^ *p* ^<^0.01; compared with cultured 5 d, ^e^ *p*<0.01; compared with cultured 6 d, ^e^ *p* ^<^0.01; compared with cultured 7 d, ^f^ *p*<0.01. **(E)** The effect of nanoformulations with different 5-FU concentrations on the intracellular drug concentration of liver cancer cells. Note: compared with 1 μg/mL, ^a^ *p*<0.01; compared with 2μ g/mL, ^b^ *p* ^<^0.01; compared with 3μg/mL, ^c^ *p*<0.01; compared with 6μg/mL, ^d^ *p*<0.01; compared with CS/5-FU, ^e^ *p* ^<^0.01; compared with 5-FU, ^f^ *p* ^<^0.01; compared with FA-CS/5-FU or Bio-CS/5-FU, ^g^ *p* ^<^0.01. **(F)** Effects of different 5-FU nanoformulations on the intracellular drug concentration of hepatoma cells after culturing for 1-8 h Note: compared with 1 h, ^a^ *p* ^<^0.01; compared with 2 h, ^b^ *p* ^<^0.01; compared with CS/5-FU, ^c^ *p* ^<^0.01; compared with 5-FU, ^d^ *p* ^<^0.01; compared with FA-CS/5-FU or Bio-CS/5-FU comparison, ^e^ *p* ^<^0.01. **(G)** The effects of various 5-FU nanoformulations on drug concentration in hepatoma cells and hepatocytes cultured for 1–8 h Note: compared with 1 h, ^a^ *p* ^<^0.01; compared with 2 h, ^b^ *p* ^<^0.01. **(H)** The impact of different 5-FU nanoformulations on the S/Q ratio after culturing for 1-8 h. Note: compared with FA-CS or Bio-CS, ^a^ *p* ^<^0.01.

From **Figures 2A-C**, it can be seen that in the FA-CS-Bio/5-FU, FA-CS/5-FU, Bio-CS/5-FU, CS/5-FU, and 5-FU groups with 5-FU administration, with the increase in concentration, the inhibition rate of tumor cells increased significantly (*p<*0.01). From **Figure 2D**, the tumor inhibition rate of the 5-FU group increased significantly with the prolongation of the action time within the time range of 1 d to 7 d (*p<*0.01). In the FA-CS-Bio/5-FU group, the tumor inhibition rates of SK-HEP-1 and SW480 cells were the highest (*p<*0.01). The mechanism was related to the dual targeting of FA-CS-Bio nanomaterials to both liver cancer and colon cancer cells. The tumor inhibition rate of SK-HEP-1 and SW480 cells in the CS/5-FU group was lower than that in the 5-FU group within 1 d~2 d (*p<*0.01). Their mechanism may be related to the fact that the concentration of 5-FU released by non-targeting of nanomaterials to tumor cells in the range of 1 d to 2 d was significantly lower than that of 5-FU, resulting in a decrease in the inhibition rate. After 3 d~6 d, the nanoparticles released 5-FU in a consistent and sustained manner, and the tumor inhibition rate was significantly higher than in the 5-FU group.

### FA-CS-Bio nanomaterials on drug concentrations in hepatocellular carcinoma cells

It can be seen from **Figure 2E** that after SK-HEP-1 cells were treated with different strategies, the concentration of 5-FU in liver cancer cells increased with the increase of the administration concentration (*p<*0.01), and the non-targeted administration groups (5-FU-SK-HEP-1 and CS/5-FU-SK-HEP-1) reached a plateau period when the concentration of 5-FU in tumor cells reached 6 µg/mL, while there was no platform period between the targeted drug groups (FA-CS-Bio/5-FU-SK-HEP-1, FA-CS/5-FU-SK-HEP-1, and Bio-CS/5-FU-SK-HEP-1). The 5-FU concentration in the targeted administration groups in liver cancer cells at each time point (FA-CS-Bio/5-FU-SK-HEP-1, FA-CS/5-FU-SK-HEP-1 and Bio-CS/5-FU-SK-HEP-1) was significantly higher than that in the non-targeted administration groups (5-FU-SK-HEP-1 and CS/5-FU-SK-HEP-1) (*p<*0.01). In the FA-CS-Bio/5-FU-SK-HEP-1 group, the concentration of 5-FU in liver cancer cells was the highest, which was significantly higher than that in the single-targeted administration mode (FA-CS/5-FU-SK-HEP-1 and Bio-CS/5-FU-SK-HEP-1) (*p<*0.01).

As can be seen from **Figure 2F**, with the increase in culture time, the concentration of 5-FU in tumor cells increased significantly (*p<*0.01). When the culture time reached 4 h, the concentration of 5-FU in tumor cells increased slowly, similar to the phenomenon of drug saturation in tumor cells. The concentration of 5-FU in tumor cells was significantly higher in the targeted drug delivery group (FA-CS-Bio/5-FU-SK-HEP-1, FA-CS/5-FU-HEP-1, and Bio-CS/5-FU-HEP-1) than in the non-targeted administration group (5-FU-SK-HEP-1 and CS/5-FU-SK-HEP-1) (*p<*0.01). The intracellular concentration of 5-FU in the FA-CS-Bio/5-FU-SK-HEP-1 group was the highest, which was significantly higher than that in the single targeting groups (FA-CS/5-FU-SK-HEP-1 and Bio-CS/5- FU-SK-HEP-1) (*p<*0.01). In the non-targeted group, the concentration of 5-FU in tumor cells in the 5-FU-SK-HEP-1 group was significantly higher than that in the CS/5-FU-SK-HEP-1 group, which may have had a slow-release effect compared to CS, so, the concentration of 5-FU was significantly lower than that of pure 5-FU within 4 h.

It can be seen from **Figure 2G** that the concentration of 5-FU in hepatoma cells of the FA-CS-Bio/5-FU-SK-HEP-1 group was significantly higher than that of the FA-CS/5-FU-SK-HEP-1 and Bio-CS/5-FU-SK-HEP-1 groups within the range of 1-8 h of cell culture (*p<*0.01). There was no significant difference in the 5-FU concentration in hepatocytes among the 5-FU, FA-CS-Bio/5-FU-SK-HEP-1, FA-CS/5-FU-SK-HEP-1 and Bio-CS/5-FU-SK-HEP-1 groups (*p* > 0.05). From **Figure 2F**, it can be seen that the S/Q level in the FA-CS-Bio/5-FU-SK-HEP-1 group was significantly higher than that in the FA-CS/5-FU-SK-HEP-1 and Bio-CS/5-FU-SK-HEP-1 groups (*p<*0.01), indicating that the targeting of FA-CS-Bio nanomaterials to liver cancer was significantly stronger than that of FA-CS and Bio-CS nanomaterials.

### FA-CS-Bio/5-FU nanoparticles on the migration ability of liver cancer cells

**Figure 3:**
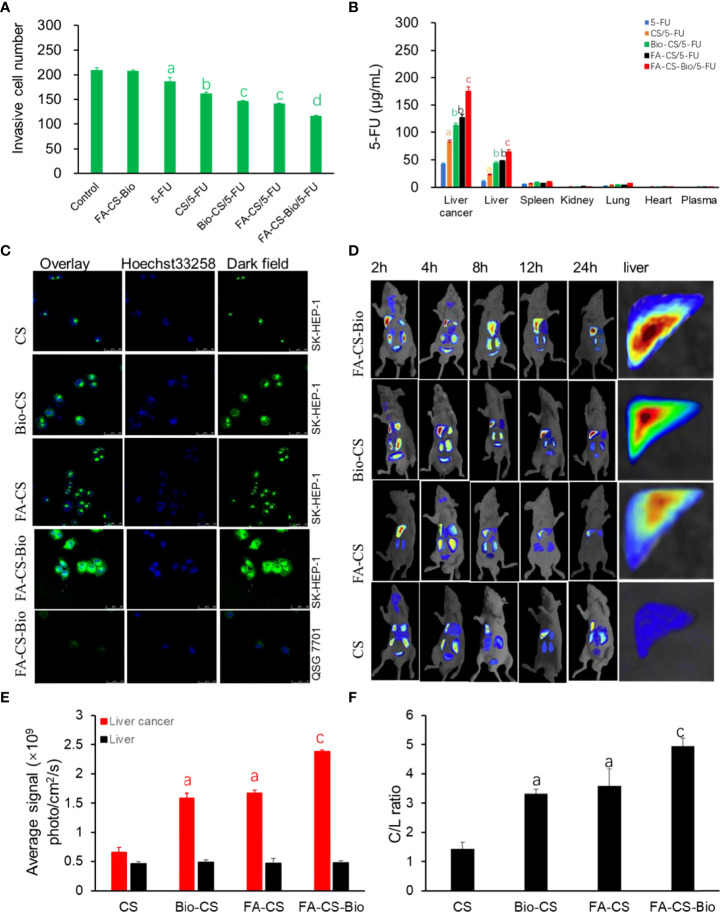
Targeting of different 5-FU nanoformulations on hepatoma cells. **(A)** Effects of different 5-FU nanoformulations on the migration of liver cancer cells. Note: compared with control, ^a^ *p* ^<^0.01; compared with 5-FU, ^b^ *p*<0.01; compared with CS/5-FU, ^c^ *p* ^<^0.01; compared with FA-CS/5-FU or Bio-CS/5-FU, ^d^ *p* ^<^0.01. **(B)** Effects of different 5-FU nano-formulations on the concentration of 5-FU in different tissues of mouse orthotopic liver cancer transplantation model. Note: compared with 5-FU, ^a^ *p* ^<^0.01; compared with CS/5-FU, ^b^ *p*<0.01; compared with FA-CS/5-FU or Bio-CS/5-FU, ^c^ *p* ^<^0.01. **(C)** Confocal detection of endocytosis of liver cancer cells by different nanomaterials. **(D)** *In vivo* imaging of different nanomaterials in a mouse orthotopic liver cancer model. **(E)** Fluorescence photon numbers of different nanomaterials in liver cancer and liver tissue. Note: compared with CS, ^a^ *p* ^<^0.01; compared with FA-CS or Bio-C, ^c^ *p* ^<^0.01. **(F)** Comparison of C/L ratios of different nanomaterials. Note: compared with CS, ap<0.01; compared with FA-CS or Bio-C, ^c^ *p* ^<^0.01.

From **Figure 3A**, the tumor cell counts were the least in the FA-CS-Bio/5-FU group (*p<*0.01). The number of tumor cells through the membrane to enter the lower chamber in the FA-CS/5-FU and Bio-CS5-FU groups was the second, and the tumor cell counts increased sequentially from CS/5-FU, 5-FU, and the control group (*p<*0.01). The control and FA-CS-Bio groups showed tumor cell counts were the most (*p<*0.01). It shows that nanomaterials carrying 5-FU can inhibit the migration of liver cancer cells. Among them, the nanomaterial FA-CS-Bio with dual liver cancer targeting properties has the strongest inhibitory effect on the migration of liver cancer cells, which is obviously stronger than that of single targeting. The nanomaterials (FA-CS and Bio-CS) can significantly improve 5-FU inhibition of hepatoma cell migration.

### 5-FU concentration of FA-CS-Bio/5-FU in liver cancer tissue

**Figure 3B** shows that the concentration of 5-FU in the liver cancer tissue of the FA-CS-Bio/5-FU group was as high as 174.94 µg/mL after 24 h, which was 4 times higher than that of the 5-FU group, which was 42.88 µg/mL. The concentration of liver cancer tissue in the/5-FU group was 83.60 µg/mL, which was 1.9 times that of the 5-FU group, which may be related to the sustained release effect of CS nanomaterials. The liver cancer tissue concentrations of the single-targeted FA-CS/5-FU group and Bio-CS were 127.20 µg/mL and 112.61 µg/mL, which were 2.9 and 2.6 times higher than that of the 5-FU group, respectively. On the basis of Bio-CS, FA-CS-Bio nanomaterials improved the targeting of liver cancer by 1.6 times. Similarly, compared with other nanomaterials, the plasma drug concentration of Bio-GC in liver cells was also significantly improved, but the increase rate was significantly lower than that in liver cancer tissues.

### Targeting effect of FA-CS-Bio/5-FU nanoparticles on liver cancer

In **Figure 3C**, through *in vitro* experiments, human normal hepatocytes QSG 7701 and human hepatoma cells SK-HEP-1 were used as models. The fluorescence intensity of the endocytosed nanoparticles was observed. It was found that FA-CS-Bio nanoparticles had the strongest green fluorescence in HepG2 cells, which was significantly higher than that of Bio-CS and FA-CS nanomaterials, and untargeted CS had the lowest fluorescence intensity. At the same time, it was found that after FA-CS-Bio treated liver cells with QSG 7701, only a small amount of green fluorescence was seen in the cells.

In order to further confirm the *in vivo* targeting of FA-CS-Bio nanomaterials, the dynamic distribution of FA-CS-Bio nanomaterials in a mouse orthotopic liver cancer transplantation model was designed. Rhodamine B was used to label the nanoparticles. From **Figures 3D, E**, it can be seen that at 24 h, the double liver cancer targeting nanomaterial FA-CS-Bio had the strongest photon number in liver cancer tissue (*p<*0.01), which was significantly higher than that of the single liver cancer targets FA-CS and Bio-CS (*p<*0.01). The photon number of the single-targeted nanomaterials was significantly higher than that of the non-targeted nanomaterials CS (*p<*0.01). There was no significant difference in the number of photons in liver tissue among dual-targeted, single-targeted, and non-targeted nanomaterials (P>0.05). The C/L ratio reflects the targeting index of liver cancer. From **Figure 3D**, it can be seen that the C/L ratio of the dual-targeted liver cancer material was the highest, and the C/L ratio of the single-targeted nanomaterials FA-CS and Bio-CS was significantly higher than that of the non-targeted nanomaterials (*p<*0.01), but there was no significant difference between single-targeted nanomaterials (*p >*0.05).

### Inhibitory of FA-CS-Bio/5-FU nanoparticles on mouse orthotopic liver cancer transplantation model

**Figure 4:**
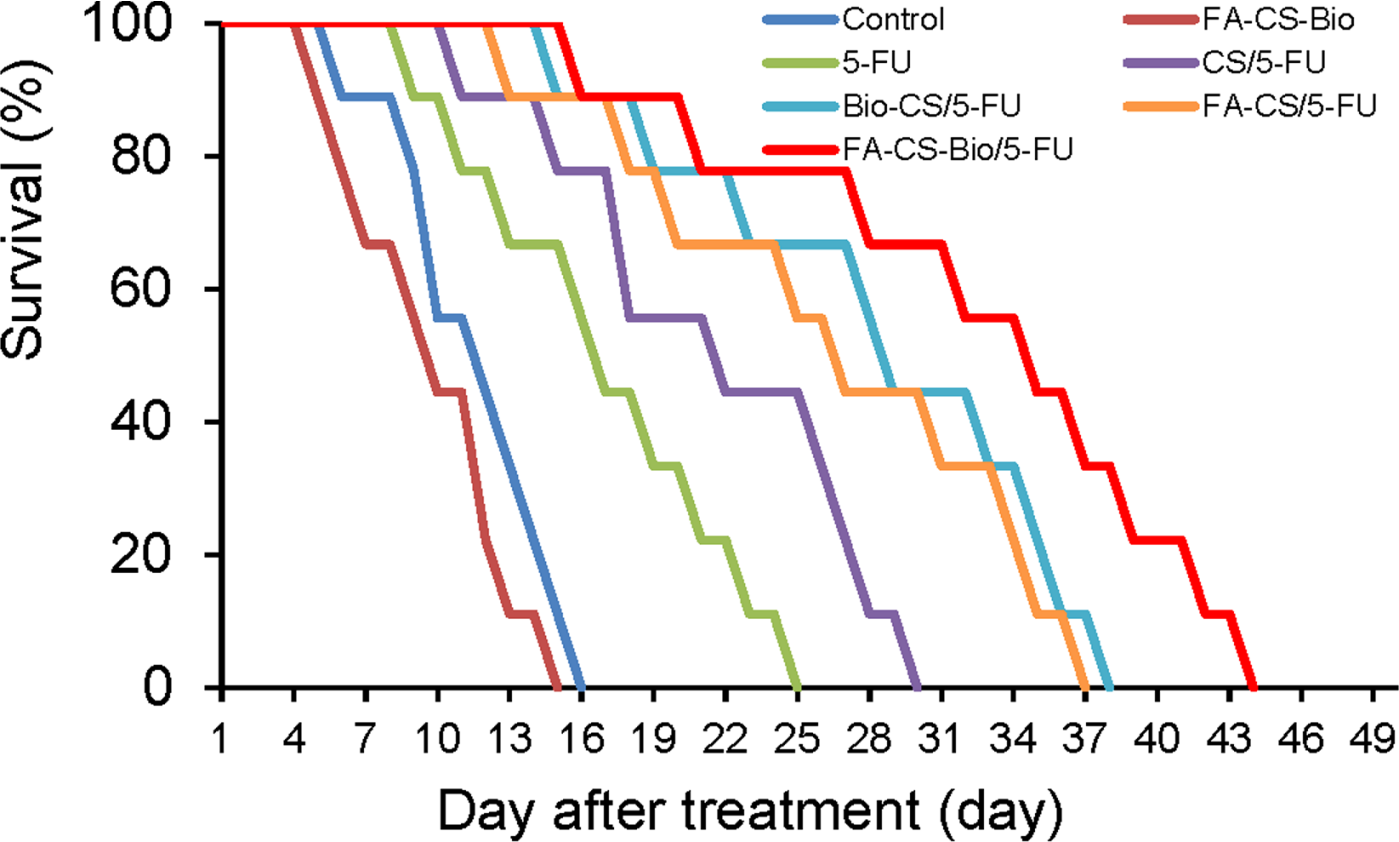
Survival analysis of different 5-FU nanoformulations in mouse orthotopic liver cancer model.

**Figure 4** shows that all mice died between 6 and 15 days after the models were formed, with a median survival time of 12 days; all mice in the FA-CS-Bio group died between the 5th and 15th day, with a median survival time of 10 days. All mice died from 9 d to 25 d, and the median survival time was 17 d; the CS/5-FU group died from 11 d to 30 d, and the median survival time was 22 d; the Bio-CS/5-FU group from 15 d to 15 d. All mice died at 38 d, with a median survival time of 28 d; the FA-CS/5-FU group died from 13 d to 37 d, with a median survival time of 27 d; and the FA-CS-Bio/5-FU group died from 16 d to 37 d. All mice died at 16 d~44 d, and the median survival time was 35 d. The Kaplan-Meier survival analysis showed that the difference was statistically significant (*χ*2 = 76.055, *p<*0.01), and the FA-CS-Bio/5-FU group had the longest survival time, which was significantly better than the single-targeted groups (Bio-CS/5-FU and FA-CS/5-FU) and non-targeting group (CS/5-FU group) (*p<*0.01), while the survival time of the single-targeting group was significantly longer than that of the non-targeting group (*p<*0.01). The inhibition of tumor-bearing mice carried by nanomaterials with 5-FU is significantly better than 5-FU, indicating that nanomaterials can help to improve the curative effect of tumor inhibition. At the same time, the nanomaterial FA-CS-Bio with dual liver cancer targeting is better than simple liver targeting. The nanomaterial Bio-CS is more beneficial for improving the efficacy of 5-FU in inhibiting liver cancer.

### Side effects of FA-CS-Bio/5-FU nanoparticles on tumor-bearing mice

In order to understand whether FA-CS-Bio nanoparticles have side effects on the body (such as liver and kidney function damage and bone marrow suppression, etc.), blood indicators in mice were detected after treatment. It can be seen from **Table 4** that there was no significant difference in serum creatinine and Hgb between before and after treatment (*p >*0.05). After treatment, the serum ALT and AST levels in the 5-FU group were significantly increased compared with the control group, and the levels of PLT, WBC, and RBC were significantly decreased, while the FA-CS-Bio and FA-CS-Bio/5-FU groups showed no significant difference in serum ALT and AST levels. It is speculated that FA-CS-Bio nanomaterials can significantly alleviate or reduce the side effects of 5-FU on tumor-bearing mice.

**Table 4:** Toxic and side effects of FA-CS-Bio/5-FU nanoparticles on tumor-bearing mice.

Groups	AST(U/L)	ALT(U/L)	Creatinine (μmol/L)	Hgb(g/L)	PLT(×10^9^/L)	WBC(×10^9^/L)	RBC(×10^12^/L)
Control	83.11 ± 12.49	34.52 ± 2.07	0.30 ± 0.04	128.44 ± 9.92	239.23 ± 11.27	7.43 ± 0.06	9.57 ± 0.27
FA-CS-Bio	85.00 ± 2.12	34.09 ± 1.90	0.28 ± 0.05	133.00 ± 12.53	243.30 ± 9.22	7.51 ± 0.26	9.55 ± 0.13
5-FU	96.42 ± 10.66^a^	47.33 ± 2.80^a^	0.28 ± 0.03	124.44 ± 11.03	205.75 ± 19.11^a^	5.22 ± 0.35^a^	7.82 ± 1.14^a^
FA-CS-Bio/5-FU	91.24 ± 6.01	33.35 ± 4.06	0.29 ± 0.03	128.43 ± 7.47	229.01 ± 13.16	7.38 ± 0.13	9.52 ± 0.21

Compared with control, ^a^p^<^0.01.

## Discussion

### Optimized synthesis of FA-CS-Bio and sustained release of FA-CS-Bio/5-FU nanoparticles

The factors affecting the synthesis of FA-CS-Bio were optimized, and it was found that the molar ratios of FA: Bio-CS and FA: EDC.HCl were 26:1 and 1:8, respectively, and the reaction time was 24 h to synthesize FA-CS-Bio. The yield was the best at room temperature, and its FA-CS-Bio yield was the highest. FA-CS-Bio was successfully synthesized by the above optimal synthesis regulation and verified by infrared spectrum and ^1^H-NMR. Compared with CS, Bio-CS showed characteristic proton peaks of Bio residues at 1.94 and 3.04 ppm in the ^1^H-NMR, indicating that the amino group of CS was successfully grafted with Bio. Compared with Bio-CS, FA-CS-Bio has characteristic proton peaks of FA at 7.2, 7.9, and 8.9 ppm, indicating that FA has been successfully grafted onto Bio-CS to form a new FA-CS-Bio nanomaterial. The DS of FA was 6.87%. Combined with the amination reaction in the infrared spectrum, it indicated that FA was successfully grafted onto Bio-CS nanomaterials.

In order to confirm whether Bio-GC nanomaterials have the characteristics of carrying chemotherapeutic drugs, a drug (5-FU) with a definite curative effect on tumor chemotherapy but relatively large toxic and side effects was selected to study the effect of Bio-GC nanomaterials carrying 5-FU on liver cancer cells. 5-FU has a broad anti-cancer spectrum and is widely used in digestive tract tumors. Like other commonly used anti-tumor drugs, it is a non-selective anti-tumor drug, which is non-specifically distributed in the body and has large toxic and side effects, which limits its clinical use to a certain extent. Therefore, scholars have carried out a lot of research on ultra-micro targeting and controlled release systems. In the early days, natural degradable materials such as serum protein (33), collagen (34) and gelatin (23) were used in the treatment of tumors. Although these materials have good biocompatibility, their widespread clinical use is limited to a certain extent due to their difficulties in preparation, high cost, uncontrollable quality, and inability to mass-produce them. In this study, the readily available biodegradable material FA-CS-Bio has the advantages of good histocompatibility and low price, and has great advantages in clinical use. Scanning electron microscopy showed that the FA-CS-Bio/5-FU nanoparticles were spherical, with a smooth surface, uniform size, good dispersion, and no agglomeration. The particle size was 80.7 nm, which was an ideal particle size. When the particle size is<30 nm, the nanoparticles are easily metabolized and cleared by the kidneys; when the particle size is >300 nm, the nanoparticles are easily cleared by the macrophage system in the liver and spleen (35). The concentration of nanoparticles entering the tissue has a greater impact. Normal vascular endothelial space is less than 20 nm, malignant space is greater than 200 nm, and some are greater than 400 nm (36). Therefore, when the particle size of the drug delivery system is between 20 nm and 300 nm, it is easier for the drug-loaded nanoparticles to enter the tumor tissue, but it is more difficult to enter the surrounding tissue. Significantly higher drug concentrations enhance tumor-suppressive effects (37, 38). The release of FA-CS-Bio/5-FU nanoparticles in SBF has three distinct release stages, namely the fast release stage, the steady release stage, and the slow-release stage. The rapid release stage may be caused by the rapid diffusion of the 5-FU physically encapsulated in the nanoparticles, and the release rate is relatively fast; the steady release stage may be caused by the hydrolysis of the amide bonds of the nanomaterials, so that the 5-FU drug encapsulated in the nanoparticle can be stabilized. The slow phase may be related to the slow and complete degradation of the nanomaterials, resulting in the slow release of 5-FU.

### FA-CS-Bio/5-FU nanoparticles inhibit liver cancer proliferation and migration by increasing intracellular 5-FU concentration

Malignant tumors are characterized by unrestricted proliferation, invasion, and metastasis, ultimately leading to death in patients with cancer (39, 40). In order to determine whether FA-CS-Bio/5-FU nanoparticles have an effect on the proliferation, invasion, and migration of hepatoma cells, we set up an experiment of FA-CS-Bio/5-FU nanoparticles on the proliferation of hepatoma cells, and found that the inhibitory effect of FA-CS-Bio/5-FU nanoparticles on liver cancer cells was positively correlated with time and drug dose, and FA-CS-Bio/5-FU nanoparticles were found to have the same inhibitory effect on liver cancer cells as colon cancer cells. This indicates that the dual cancer-targeting FA-CS-Bio nanomaterials have stronger targeting to liver cancer and colon cancer cells than the single liver cancer-targeting nanomaterials (FA-CS and Bio-CS), which may be related to the nanomaterials mediating intracellular 5-FU concentration being higher, which showed stronger inhibition of the proliferation of liver cancer cells. In the liver cancer migration experiment, it was found that the tumor cells in the upper chamber of the Trans well in the FA-CS-Bio/5-FU group had the largest number of apoptosis, which was significantly higher than that in the FA-CS/5-FU and Bio-CS/5-FU groups, while the cells in the lower compartment showed the opposite change, that is, the smallest number of cells. This indicates that FA-CS-Bio/5-FU nanoparticles can make more 5-FU enter into liver cancer cells through biotin and folate receptors, and 5-FU regulates intracellular apoptosis signals (41) so as to achieve an inhibitory effect on liver cancer migration. This study found that FA-CS-Bio can significantly increase the concentration of 5-FU in liver cancer cells compared with single-targeted and non-targeted nanomaterials and found that FA-CS-Bio did not increase the concentration of 5-FU in liver cells. Non-targeted nanomaterials carry 5-FU to treat liver cancer cells. With the increase of 5-FU administration concentration, a plateau phase occurs after the intracellular 5-FU concentration rises, while liver cancer-targeted nanomaterials carry 5-FU. In the treatment of liver cancer, the increase of intracellular 5-FU concentration has no obvious plateau, which may be related to the entry of 5-FU into the cell through the receptor by the targeted nanomaterials. Within 1-4 h of culture, the concentration of 5-FU in the cells increased significantly, while the increase in the concentration of 5-FU in tumor cells slowed down significantly after more than 4 h. In this study, it was found that the S/Q mediated by FA-CS-Bio nanomaterials was significantly higher than that of Bio-CS, indicating that the grafting of FA into Bio-CS significantly improved the targeting of liver cancer cells, thereby increasing the intracellular 5-FU concentration.

### Targeting effect of FA-CS-Bio nanomaterials on liver cancer *in vitro* and *in vivo*

In the laser confocal experiment, it was also found that FA-CS-Bio nano-mediated endocytosis was significantly stronger than that of single-targeted and non-targeted nanomaterials, and it was found that after FA-CS-Bio was treated with liver cells, only a small amount of intracellular green fluorescence. It shows that FA-CS-Bio nanomaterials have an obvious targeting effect on liver cancer cells, and the mechanism is related to the active targeting effect mediated by ligand receptors. After grafting FA into the Bio-CS material, the endocytosis of the nanomaterials by hepatoma cells was significantly enhanced, and the intracellular green fluorescence was significantly enhanced. Together with the FA-CS-Bio nanomaterials, the nanomaterials also possessed FA that bound to the receptors of hepatoma cells. It is related to the two ligands of Bio, which have stronger targeting for liver cancer. In addition, FA-CS-Bio nanomaterials with two ligands (FA and Bio) for liver cancer were found in the dynamic distribution experiment of living mice, which made the fluorescence signal the strongest in liver cancer tissue and had the maximum number of fluorescence photons per unit area. The mechanism is related to the characteristics of the FA-CS-Bio nanomaterial itself: it has both FA and Bio liver cancer targeting ligands. After the nanomaterial is injected into the tail vein, the nanomaterial first enters the liver through the portal vein, so FA-CS-Bio materials have the ability to aggregate nanomaterials into liver cancer tissue. In terms of cellular mechanism, since liver cancer cells have both FA and Bio receptors, they have dual liver cancer targeting to liver cancer cells. The concentration of dual-targeted nanomaterials on tumor cells is significantly stronger than that of single-targeted nanomaterials (42), so it is inferred that FA-CS-Bio material mediates the significantly higher concentration of 5-FU in liver cancer tissue than in FA-CS and Bio-CS nanomaterials, as confirmed in the mouse orthotopic liver cancer transplantation model. This study found that the concentration of 5-FU in the liver cancer tissue of the FA-CS-Bio/5-FU group increased by 4 times at 24 h compared with the 5-FU group and increased by 1.6 times compared with the single-targeted Bio-CS/5-FU group. Similarly, compared with other nanomaterials, FA-CS-Bio can also improve the blood drug concentration of liver cells, but the increase is significantly lower than that of liver cancer tissue. It shows that the modified FA-CS-Bio nanomaterial has a significant improvement in the targeting of liver cancer compared with Bio-CS. The concentration of 5-FU in liver cancer tissue is the highest, followed by liver tissue, but 5-FU in liver tissue has the highest concentration. The concentration was significantly lower than that of Bio-CS, indicating that compared with Bio-CS, FA-CS-Bio nanomaterials can increase the concentration of 5-FU in liver cancer tissue and reduce the concentration of 5-FU in normal liver tissue.

### FA-CS-Bio/5-FU nanoparticles can prolong the survival of tumor-bearing mice and reduce the side effects of 5-FU

In order to further understand the efficacy of Bio-GC/5-FU nanoparticles in inhibiting liver cancer *in vivo*, in this study, a mouse liver cancer orthotopic transplantation model was successfully constructed. After treatment with various nanotherapeutic strategies, survival analysis found that the FA-CS-Bio/5-FU group had the longest survival, which was significantly longer than the FA-CS/5-FU, Bio-CS/5-FU, CS/5-FU, and 5-FU groups. It shows that the CS/5-FU nanoparticles with sustained-release effect have a significantly higher efficacy than 5-FU in the treatment of liver cancer. The results showed that liver cancer-targeted nanomaterials can improve the efficacy of 5-FU in the treatment of orthotopic liver cancer models, and FA-CS-Bio nanomaterials help to increase the intracellular 5-FU concentration, which is expressed as the killing effect on hepatoma cells. It is known that the side effects of 5-FU are mainly bone marrow suppression and liver function damage, among which the reduction of WBC and PLT is the most obvious. In this study, it was found through animal experiments that compared with the control group, the levels of ALT and AST in the 5-FU group were significantly increased, and the levels of PLT, WBC, and RBC were significantly decreased, while there were no significant changes in the FA-CS-Bio and FA-CS-Bio/5-FU groups in those indicators. It shows that 5-FU can damage liver function and inhibit bone marrow, while FA-CS-Bio nanoparticles can alleviate the damage of 5-FU to liver cells and inhibit bone marrow hematopoiesis. Therefore, FA-CS-Bio nanoparticles can not only improve the targeting of 5-FU to liver cancer but also alleviate the liver damage and bone marrow suppression caused by chemotherapy drugs.

## Conclusions

- The synthesis of FA-CS-Bio nanomaterials has been optimized. When FA: Bio-CS is 26:1, FA: EDC.HCl is 1:8, the reaction time is 24 h, and the reaction is carried out at room temperature, the highest yield of FA-CS-Bio was 23.67%, and the synthesis of FA-CS-Bio nanomaterials was confirmed by Fourier transform infrared spectroscopy and hydrogen nuclear magnetic resonance spectroscopy.
- Compared with Bio-CS, FA-CS-Bio nanomaterials can significantly improve the targeting of liver cancer and the concentration of drugs in liver cancer cells in both laser confocal experiments and *in vivo* mouse experiments. The concentration of 5-FU in liver cancer tissue in FA-CS-Bio/5-FU nanoparticles was increased by 1.6 times compared with the Bio-CS/5-FU nanoparticles.

 iii. FA-CS-Bio/5-FU nanoparticles have obvious sustained-release effects, which are in the rapid release stage, steady release stage, and slow release stage. FA-CS-Bio/5-FU nanoparticles can inhibit the proliferation of hepatoma cells in a dose-and time-dependent manner and have a significant inhibitory effect on the migration of hepatoma cells.

 iv. Compared with Bio-CS/5-FU nanoparticles, FA-CS-Bio/5-FU nanoparticles can significantly prolong the survival time of an orthotopic liver cancer transplantation model in mice, and FA-CS-Bio nanomaterials can significantly alleviate the toxic and side effects caused by 5-FU.

## Data availability statement

The raw data supporting the conclusions of this article will be made available by the authors, without undue reservation.

## Ethics statement

All animals were treated following the protocol approved by the Institutional Animal Care and Use Committee at the Shanghai Tianyou Hospital.

## Author contributions

MC and DD conceived and designed the experiments. MC performed this experimental work and analyzed the data. MC and DD participated in the experiments. All authors read and approved the final manuscript.

## Funding

This study was financially supported by Scientific Research Project of Shanghai Municipal Health Commission (Grant No. 201940430).

## Acknowledgments

We would like to acknowledge the School of Life Sciences and Technology of Tongji University for providing technological support.

## Conflict of interest

The authors declare that the research was conducted in the absence of any commercial or financial relationships that could be construed as a potential conflict of interest.

## Publisher’s note

All claims expressed in this article are solely those of the authors and do not necessarily represent those of their affiliated organizations, or those of the publisher, the editors and the reviewers. Any product that may be evaluated in this article, or claim that may be made by its manufacturer, is not guaranteed or endorsed by the publisher.

## Abbreviations

CS, chitosan; Bio, Biotin`; Bio-CS, galactosylated chitosan; FA, folic acid; ASGPR, asialoglycoprotein receptor; FA-CS-Bio, glycosylated chitosan modified by FA; 5-FU; fluorouracil; PY, product yield; EDC, 1-ethyl-3-(3-dimethylaminopropyl) carbodiimide hydrochloride; DMAc, dimethylacetamide; DMF, dimethylformamide; DMSO, dimethyl sulfoxide; DS, degree of substitution; HAC, acetic acid; SBF, standard body fluid; RBITC, Rhodamine B Isothiocyanate; ALT, alanine aminotransferase; WBC, white blood cells; RBC, red blood cells; Hgb, hemoglobin; PLT, platelets; ANOVA, one-way analysis of variance; LSD, least significance difference.

## References

[1] Elgadir MA Uddin MS Ferdosh S Adam A Chowdhury A Sarker M. Impact of chitosan composites and chitosan nanoparticle composites on various drug delivery systems: a review. J Food Drug Anal (2015) 23:619–29. doi: 10.1016/j.jfda.2014.10.008 PMC9345468 28911477

[2] Chen YY Lin YJ Huang WT Hung CC Lin HY Tu YC. Demethoxycurcumin-loaded chitosan nanoparticle downregulates dna repair pathway to improve cisplatin-induced apoptosis in non-small cell lung cancer. Molecules (2018) 23(12): 3217. doi: 10.3390/molecules23123217 PMC6320861 30563166

[3] Pramanik A Laha D Pramanik P Karmakar P. A novel drug "copper acetylacetonate" loaded in folic acid-tagged chitosan nanoparticle for efficient cancer cell targeting. J Drug Targeting (2014) 22:23–33. doi: 10.3109/1061186X.2013.832768 23987131

[4] Rao W Wang H Han J Zhao S Dumbleton J Agarwal P. Chitosan-decorated doxorubicin-encapsulated nanoparticle targets and eliminates tumor reinitiating cancer stem-like cells. ACS Nano. (2015) 9:5725–40. doi: 10.1021/nn506928p 26004286

[5] Huo J. Effects of chitosan nanoparticle-mediated braf sirna interference on invasion and metastasis of gastric cancer cells. Artif Cells Nanomed Biotechnol (2016) 44:1232–5. doi: 10.3109/21691401.2015.1019666 25794798

[6] Guan M Zhou Y Zhu QL Liu Y Bei YY Zhang XN. N-trimethyl chitosan nanoparticle-encapsulated lactosyl-norcantharidin for liver cancer therapy with high targeting efficacy. Nanomedicine-Uk (2012) 8:1172–81. doi: 10.1016/j.nano.2012.01.009 22321383

[7] Marques AC Costa PJ Velho S Amaral MH. Functionalizing nanoparticles with cancer-targeting antibodies: a comparison of strategies. J Control Release (2020) 320:180–200. doi: 10.1016/j.jconrel.2020.01.035 31978444

[8] Zhong Y Meng F Deng C Zhong Z. Ligand-directed active tumor-targeting polymeric nanoparticles for cancer chemotherapy. Biomacromolecules (2014) 15:1955–69. doi: 10.1021/bm5003009 24798476

[9] Jain A Cheng K. The principles and applications of avidin-based nanoparticles in drug delivery and diagnosis. J Control Release (2017) 245:27–40. doi: 10.1016/j.jconrel.2016.11.016 27865853 PMC5222781

[10] Shiraishi T Eysturskarth J Nielsen PE. Modulation of mdm2 pre-mrna splicing by 9-aminoacridine-pna (peptide nucleic acid) conjugates targeting intron-exon junctions. BMC Cancer (2010) 10:342. doi: 10.1186/1471-2407-10-342 20591158 PMC2910690

[11] Heo DN Yang DH Moon HJ Lee JB Bae MS Lee SC. Gold nanoparticles surface-functionalized with paclitaxel drug and biotin receptor as theranostic agents for cancer therapy. Biomaterials (2012) 33:856–66. doi: 10.1016/j.biomaterials.2011.09.064 22036101

[12] Cheng M Zhu W Li Q Dai D Hou Y. Anti-cancer efficacy of biotinylated chitosan nanoparticles in liver cancer. Oncotarget (2017) 8:59068–85. doi: 10.18632/oncotarget.19146 PMC5601715 28938619

[13] Zhou N Zan X Wang Z Wu H Yin D Liao C. Galactosylated chitosan-polycaprolactone nanoparticles for hepatocyte-targeted delivery of curcumin. Carbohydr Polym (2013) 94:420–9. doi: 10.1016/j.carbpol.2013.01.014 23544558

[14] Mishra D Jain N Rajoriya V Jain AK. Glycyrrhizin conjugated chitosan nanoparticles for hepatocyte-targeted delivery of lamivudine. J Pharm Pharmacol (2014) 66:1082–93. doi: 10.1111/jphp.12235 24641311

[15] Nie J Ta N Liu L Shi G Kang T Zheng Z. Activation of camkii *via* er-stress mediates coxsackievirus b3-induced cardiomyocyte apoptosis. Cell Biol Int (2020) 44:488–98. doi: 10.1002/cbin.11249 31631456

[16] Kakimoto S Moriyama T Tanabe T Shinkai S Nagasaki T. Dual-ligand effect of transferrin and transforming growth factor alpha on polyethyleneimine-mediated gene delivery. J Control Release (2007) 120:242–9. doi: 10.1016/j.jconrel.2007.05.001 17574290

[17] Hu Y Wang Y Jiang J Han B Zhang S Li K. Preparation and characterization of novel perfluorooctyl bromide nanoparticle as ultrasound contrast agent *via* layer-by-layer self-assembly for folate-receptor-mediated tumor imaging. BioMed Res Int (2016) 2016:6381464. doi: 10.1155/2016/6381464 27652265 PMC5019893

[18] Samadian H Hosseini-Nami S Kamrava SK Ghaznavi H Shakeri-Zadeh A. Folate-conjugated gold nanoparticle as a new nanoplatform for targeted cancer therapy. J Cancer Res Clin Oncol (2016) 142:2217–29. doi: 10.1007/s00432-016-2179-3 PMC11819103 27209529

[19] Tang Q Chen D. Study of the therapeutic effect of 188re labeled folate targeting albumin nanoparticle coupled with cis-diamminedichloroplatinum cisplatin on human ovarian cancer. BioMed Mater Eng. (2014) 24:711–22. doi: 10.3233/BME-130859 24211956

[20] Battogtokh G Ko YT. Mitochondrial-targeted photosensitizer-loaded folate-albumin nanoparticle for photodynamic therapy of cancer. Nanomedicine-Uk (2017) 13:733–43. doi: 10.1016/j.nano.2016.10.014 27815176

[21] Yan C Gu J Jing H Taishi J Lee RJ. Tat-tagged and folate-modified n-succinyl-chitosan (tat-suc-fa) self-assembly nanoparticle for therapeutic delivery ogx-011 to a549 cells. Mol Pharm (2017) 14:1898–905. doi: 10.1021/acs.molpharmaceut.6b01167 28464609

[22] Yang CL Chen JP Wei KC Chen JY Huang CW Liao ZX. Release of doxorubicin by a folate-grafted, chitosan-coated magnetic nanoparticle. Nanomater (Basel) (2017) 7(4):85. doi: 10.3390/nano7040085 PMC5408177 28406429

[23] Khan SA. Mini-review: opportunities and challenges in the techniques used for preparation of gelatin nanoparticles. Pak J Pharm Sci (2020) 33:221–8. doi: 10.36721/PJPS.2020.33.1.REG.221-228.1 32122852

[24] Weisspflog J Vehlow D Muller M Kohn B Scheler U Boye S. Characterization of chitosan with different degree of deacetylation and equal viscosity in dissolved and solid state - insights by various complimentary methods. Int J Biol Macromol (2021) 171:242–61. doi: 10.1016/j.ijbiomac.2021.01.010 33418043

[25] Cheng M Ma D Zhi K Liu B Zhu W. Synthesis of biotin-modified galactosylated chitosan nanoparticles and their characteristics *in vitro* and *in vivo* . Cell Physiol Biochem (2018) 50:569–84. doi: 10.1159/000494169 30308481

[26] Pi C Wei Y Yang H Zhou Y Fu J Yang S. Development of a hplc method to determine 5-fluorouracil in plasma: application in pharmacokinetics and steady-state concentration monitoring. Int J Clin Pharmacol Ther (2014) 52:1093–101. doi: 10.5414/CP202120 25161161

[27] Lakkakula JR Matshaya T Krause RW. Cationic cyclodextrin/alginate chitosan nanoflowers as 5-fluorouracil drug delivery system. Mater Sci Eng C Mater Biol Appl (2017) 70:169–77. doi: 10.1016/j.msec.2016.08.073 27770878

[28] Lo CW Chan C Yu J He M Choi C Lau J. Development of cd44e/s dual-targeting dna aptamer as nanoprobe to deliver treatment in hepatocellular carcinoma. Nanotheranostics (2022) 6:161–74. doi: 10.7150/ntno.62639 PMC8671951 34976591

[29] Cheng M Li Q Wan T Hong X Chen H He B. Synthesis and efficient hepatocyte targeting of galactosylated chitosan as a gene carrier *in vitro* and *in vivo* . J BioMed Mater Res B Appl Biomater (2011) 99:70–80. doi: 10.1002/jbm.b.31873 21656667

[30] Yi J Chen S Yi P Luo J Fang M Du Y. Pyrotinib sensitizes 5-fluorouracil-resistant her2(+) breast cancer cells to 5-fluorouracil. Oncol Res (2020) 28:519–31. doi: 10.3727/096504020X15960154585410 PMC7751227 32727638

[31] Chen Y Xiang M Wang Z. Application of novel hollow carbon nanosphere drug-loading system in chemotherapy of esophageal squamous cell carcinoma. J Nanosci Nanotechnol (2021) 21:814–23. doi: 10.1166/jnn.2021.18677 33183412

[32] Chen Z Dean-Ben XL Liu N Gujrati V Gottschalk S Ntziachristos V. Concurrent fluorescence and volumetric optoacoustic tomography of nanoagent perfusion and bio-distribution in solid tumors. BioMed Opt Express (2019) 10:5093–102. doi: 10.1364/BOE.10.005093 PMC6788589 31646032

[33] An FF Zhang XH. Strategies for preparing albumin-based nanoparticles for multifunctional bioimaging and drug delivery. Theranostics (2017) 7:3667–89. doi: 10.7150/thno.19365 PMC5667340 29109768

[34] Slowinska K. Cross-linked collagen gels using gold nanoparticles. Methods Mol Biol (2018) 1798:203–12. doi: 10.1007/978-1-4939-7893-9_16 PMC6487855 29868962

[35] Jeannot V Gauche C Mazzaferro S Couvet M Vanwonterghem L Henry M. Anti-tumor efficacy of hyaluronan-based nanoparticles for the co-delivery of drugs in lung cancer. J Control Release (2018) 275:117–28. doi: 10.1016/j.jconrel.2018.02.024 29474960

[36] Iacovita C Florea A Scorus L Pall E Dudric R Moldovan AI. Hyperthermia, cytotoxicity, and cellular uptake properties of manganese and zinc ferrite magnetic nanoparticles synthesized by a polyol-mediated process. Nanomater (Basel) (2019) 9(10):1489. doi: 10.3390/nano9101489 PMC6835619 31635415

[37] Cai Z Chattopadhyay N Yang K Kwon YL Yook S Pignol JP. (111)in-labeled trastuzumab-modified gold nanoparticles are cytotoxic *in vitro* to her2-positive breast cancer cells and arrest tumor growth *in vivo* in athymic mice after intratumoral injection. Nucl Med Biol (2016) 43:818–26. doi: 10.1016/j.nucmedbio.2016.08.009 27788375

[38] Pandey SK Patel DK Maurya AK Thakur R Mishra DP Vinayak M. Controlled release of drug and better bioavailability using poly(lactic acid-co-glycolic acid) nanoparticles. Int J Biol Macromol (2016) 89:99–110. doi: 10.1016/j.ijbiomac.2016.04.065 27112980

[39] Liu YF Yang A Liu W Wang C Wang M Zhang L. Nme2 reduces proliferation, migration and invasion of gastric cancer cells to limit metastasis. PloS One (2015) 10:e115968. doi: 10.1371/journal.pone.0115968 PMC4336288 25700270

[40] Li J Jia L Ma ZH Ma QH Yang XH Zhao YF. Axl glycosylation mediates tumor cell proliferation, invasion and lymphatic metastasis in murine hepatocellular carcinoma. World J Gastroenterol (2012) 18:5369–76. doi: 10.3748/wjg.v18.i38.5369 PMC3471105 23082053

[41] Gordon SR Climie M Hitt AL. 5-fluorouracil interferes with actin organization, stress fiber formation and cell migration in corneal endothelial cells during wound repair along the natural basement membrane. Cell Motil Cytoskeleton (2005) 62:244–58. doi: 10.1002/cm.20099 16283632

[42] Jing F Li J Liu D Wang C Sui Z. Dual ligands modified double targeted nano-system for liver targeted gene delivery. Pharm Biol (2013) 51:643–9. doi: 10.3109/13880209.2012.761245 23527957

